# Basic biology and roles of CEBPD in cardiovascular disease

**DOI:** 10.1038/s41420-025-02357-4

**Published:** 2025-03-14

**Authors:** Tongjun Li, Shaoling Lin, Yingyin Zhu, Dewei Ye, Xianglu Rong, Lexun Wang

**Affiliations:** Guangdong Metabolic Diseases Research Center of Integrated Chinese and Western Medicine; Key Laboratory of Glucolipid Metabolic Disorder, Ministry of Education of China; Guangdong Key Laboratory of Metabolic Disease Prevention and Treatment of Traditional Chinese Medicine; Institute of Chinese Medicine, Guangdong Pharmaceutical University, Guangzhou, Guangdong Province China

**Keywords:** Cardiovascular diseases, Molecular biology

## Abstract

CCAAT/enhancer-binding protein delta (CEBPD), as an evolutionarily conserved protein in mammals, belongs to the CEBP transcription factor family, which modulates many biological processes. The diversity of CEBPD functions partly depends on the cell type and cellular context. Aberrant CEBPD expression and activity are associated with multiple organ diseases, including cardiovascular diseases. In this review, we describe the basic molecular biology of CEBPD to understand its expression regulation, modifications, and functions. Here, we summarize the recent advances in genetically modified animals with CEBPD. Finally, we discuss the contribution of CEBPD to cardiovascular diseases and highlight the strategies for developing novel therapies targeting CEBPD.

## Facts

- CEBPD has transcription-dependent and transcription-independent functions.
- The expression and activity of CEBPD are regulated at multiple levels.
- Elevated CEBPD is involved in the progression of cardiovascular diseases in various ways.
- New approaches targeting CEBPD for the treatment of cardiovascular diseases have gained significant interest.

## Open Questions

- The non-transcriptional function of transcription factor CEBPD requires further investigation.
- Although evidence shows that CEBPD levels are elevated in cardiovascular disease, the clear expression patterns of CEBPD in cardiac and vascular tissues during different disease states are still unknown.
- In the heart and vascular tissues, functional substitutions that exist between other CEBP family members and CEBPD remain to be clarified.

## Introduction

Human CCAAT/enhancer-binding protein delta (CEBPD), formerly known as the nuclear factor for interleukin 6 beta (IL-6β), is a 269-amino acid protein encoded by an intronless gene CEBPD. It belongs to the CEBP family of the basic-leucine zipper (bZIP) class transcription factors [1]. All members of this family (alpha, beta, delta, epsilon, gamma, zeta) share a highly conserved C-terminal region, comprising a basic amino acid-rich DNA-binding motif followed by a bZIP domain responsible for dimerization, whereas their N-termini is diverse [1, 2]. These transcription factors regulate various physiological processes, including the cell cycle, cellular differentiation, metabolism, and immune responses. Several reviews have been published on CEBPs in healthy individuals and those with various diseases [1–7]. In addition, many studies have shown that CEBPD plays crucial roles in tumorigenesis, inflammatory responses, and neurological diseases, and excellent reviews on these effects being published over the past decade [7–11]. Research has shown the following: (1) CEBPD acts as an oncogene or cancer suppressor, depending on the cancer type and microenvironments; (2) CEBPD regulates the inflammatory process by promoting or inhibiting pro-inflammatory pathways in response to different inflammatory stimuli; and (3) CEBPD is involved in neuroinflammation and some diseases of the central nervous system.

Recent studies have shown that CEBPD is vital in various physiological processes. In parturition, elevated CEBPD in amniotic fibroblast upregulates its target genes cyclooxygenase-2 (COX-2) and hydroxysteroid 11-β dehydrogenase 1 (HSD11β1), which leads to an increase in their products prostaglandin E2 (PGE2) and cortisol levels, accelerating the labor process [12]. A recent study showed that hypothalamic CEBPD is a semilunar transcription factor that directly regulates the expression of gonadotropin-releasing hormone 1, mediating the lunar-synchronized beach-spawning behavior of grass puffers [13]. Furthermore, CEBPD is involved in bone formation and maintenance of normal skin structure and function by regulating osteoblast and keratinocyte differentiation, respectively [14, 15]. Moreover, CEBPD acts as a substrate adaptor that binds to Fanconi anemia group protein D2 (FANCD2) and importin 4 to augment the nuclear import of FANCD2 for DNA repair, suggesting that CEBPD has important transcription-independent functions [16].

In addition, accumulating evidence has shown that CEBPD plays a vital role in cardiovascular diseases. Upregulation of CEBPD in the epicardial cells of the heart caused by myocardial infarction (MI) promotes heart regeneration in zebrafish [17]. In mice, elevated levels of epicardial CEBPD caused by MI facilitate neutrophil infiltration into the injured areas, exacerbating MI-induced mouse cardiac damage [18]. Furthermore, elevated CEBPD upregulates connective tissue growth factor (CTGF) expression in cardiomyocytes to activate cardiac fibroblasts, leading to cardiac fibrosis [19]. A recent study showed that CEBPD protein co-localizes with macrophages in human and mouse atherosclerotic plaques [20]. Further studies have shown that CEBPD accelerates atherosclerosis progression by promoting the conversion of macrophages and vascular smooth muscle cells (VSMCs) into foam cells [20, 21]. In addition, CEBPD levels are significantly increased in the peripheral monocytes of patients with MI compared with healthy individuals, and patients with acute MI have higher CEBPD level in peripheral blood mononuclear cells than those with stable coronary artery disease (CAD) [22, 23]. These studies indicate that elevated CEBPD levels are involved in the onset and progression of cardiovascular diseases.

This review discusses the basic molecular and biological properties of CEBPD, and its crucial roles in cardiovascular diseases. We also discuss novel findings and future directions for disease therapies based on the targeting of CEBPD.

## Cebpd biological features

### CEBPD gene and protein

The National Center for Biotechnology Information database has 719 records of CEBPD genes, including 718 in chordates and 1 in arthropods (up to 30th December 2024). However, the CEBPD gene has not been documented in viruses, bacteria, fungi, plants, or most invertebrates. This suggests that it is a late-emerging gene in evolution and may be associated with the complexity of organisms and the diversity of environmental stimuli. CEBPD is an intron-less gene that produces a single messenger RNA (mRNA) in all species except in *Xenopus laevis* [24]. The expression level of the CEBPD is typically low in most cells under normal physiological conditions, and its mRNA is highly unstable, with a half-life of 35-40 min [25, 26].

**Fig. 1:**
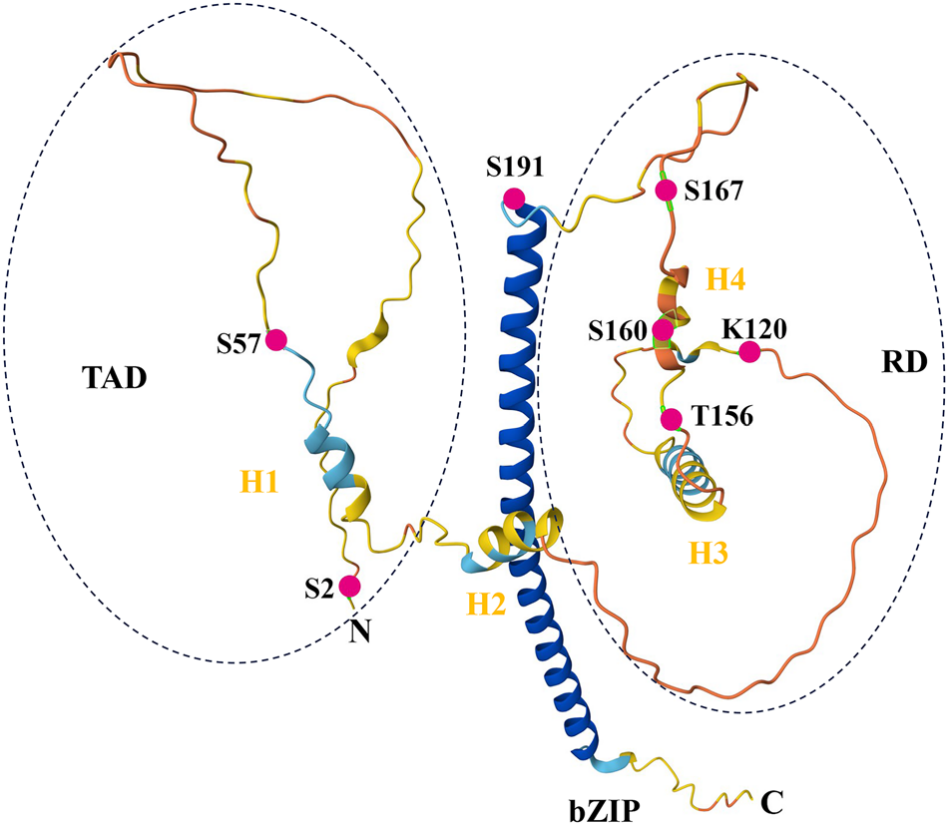
3D structure and key amino acid residues of CEBPD protein. The structure of CEBPD as predicted by AlphaFold. The N- and C-termini are indicated. The basic-leucine zipper (bZIP) domain appears as the vertical coils in blue. Besides, CEBPD contains the N-terminal transcriptional activation domain (TAD) and the regulatory domain (RD) in the middle. There are two helices (H1 and H2) in the TAD and two helices (H3 and H4) in the RD. S: Serine; T: Threonine; K: Lysine.

Human CEBPD mRNA can be translated into a single protein of approximately 28 kilo-Dalton with 269 amino acids. This protein contains an N-terminal transcriptional activation domain (TAD), a regulatory domain (RD), and a C-terminal bZIP domain. There were two helices (H1 and H2) in the TAD and two helices (H3 and H4) in the RD (Fig. 1). The H1 helix contributes to most CEBPD activation [27]. The TAD domain mediates trans-activation or trans-repression and distinguishes CEBPD from other CEBP members [8]. CEBPD binds directly to cyclic adenosine monophosphate-response element binding protein (CREB) through helices H1 and H2 in the TAD domain [28] and to mitogen-activated protein kinase 14 (p38) and E1A binding protein p300 (p300) through the H2 helix in the TAD domain [29]. In addition, CEBPD binds to the protein inhibitor of activated signal transducer and activator of transcription (STAT) 4 through its TAD domain to mediate CEBPD translocation from nuclear foci to the nuclear periphery [30]. Serine (Ser) 2, Ser 57, and Ser 62 in the TAD domain are key sites that regulate the CEBPD transcriptional activity [1, 27, 31]. The RD domain contains several key amino acid residues that regulate CEBPD function, including lysine (Lys) 120, threonine (Thr) 156, Ser 160, Ser 167, and Thr 171 [1, 10, 32]. Additionally, CEBPD interacts with siah E3 ubiquitin protein ligase 2 (SIAH2), FANCD2, and F-box and WD repeat domain containing 7 (FBXW7) through the RD domain [10, 16, 33]. The bZIP domain of CEBPD is highly conserved and comprises a basic DNA-binding domain that also serves as a nuclear localization signal, a leucine zipper domain for heterotypic or homotypic dimerization, and a tail domain that is required for interaction with other proteins [1, 8]. Ser 191 is a crucial residue in the bZIP domain for the CEBPD function [27] (Fig. 1).

### Regulation of CEBPD expression

Previous studies have shown that CEBPD expression is regulated at three levels: transcription factors, mRNA stability, and protein degradation.

Transcription factors that bind to the *CEBPD* promoter not only directly upregulate but also repress its expression. For example, STAT3 can bind to the *CEBPD* promoter and directly upregulate its expression in muscle cells and cardiomyocytes [34, 35]. In contrast, sex-determining region-box transcription factor 9 binds to the same promoter and inhibits its expression in mouse embryo fibroblasts [36]. Transcription factors regulate CEBPD expression by forming complexes with other proteins via epigenetic modifications. After binding to the promoter, jun dimerization protein 2 recruits histone deacetylase 3 (HDAC3) to inhibit the acetylation of histone H3 at this promoter, thereby repressing *CEBPD* expression [37].

In mRNA stability, the RNA binding protein Hu antigen R can bind to the 3’-untranslated region (3’-UTR) of CEBPD mRNA and stabilize it, thereby increasing its expression in macrophages [38]. In addition, pumilio RNA binding family member 2 binds to CEBPD mRNA and promotes its degradation, thereby inhibiting its expression [39]. MicroRNAs (miRNAs) are a class of small noncoding RNAs that regulate gene expression through degradation or inhibition of specific mRNA targets by binding to their 3’-UTR. MiRNA let-7c, let-7-5p, and miR-22-3p directly inhibit CEBPD expression by promoting its mRNA degradation [40–42]. In protein degradation, kelch-like family member 9, constitutive photomorphogenic 1 E3 ubiquitin ligase (COP1), SIAH2, and tripartite motif containing 26 (TRIM26) enhance the ubiquitination of CEBPD, leading to increased ubiquitin-mediated degradation and reduced protein levels [43–45].

CEBPD regulates its expression through positive and negative feedback loops. For instance, CEBPD can not only directly bind to its gene promoter alone to upregulate its expression. It can also form a complex with bromodomain-containing 4 (BRD4) or forkhead box O1 to enhance its gene transcription and increase its protein levels in VSMCs and skeletal muscle cells, respectively [46–48]. In self-negative feedback, CEBPD can upregulate the expression of its target genes miR-744, miR-3154, and miR-3162, which form a complex with argonaute 2, yin yang 1, polycomb group protein, and DNA methyltransferase to target cytosine-guanine islands in the 5’-flanking regions of the CEBPD gene, leading to its gene inactivation through epithetical modification [49]. Detailed upstream regulators of CEBPD are listed in Table 1.

**Table 1:** The upstream regulators of CEBPD expression.

Type of regulation	Regulators	Effects	Cells	References
**Transcriptional regulation**	ATF3	Binds to the promoter of *CEBPD* gene to **repress** its expression	Macrophages	[123]
	ATF4	Binds to the promoter of *CEBPD* gene to **upregulate** its expression	Adipocytes	[124]
	BRD4	Forms a complex with CEBPD to binds the promotor of *CEBPD* gene for **upregulating** its expression	Vascular smooth muscle cells	[47]
	CEBPD	Binds to the promotor of *CEBPD* gene to **upregulate** its expression	Macrophages, Renal fibroblasts	[46, 123]
	c-JUN	Binds to the promotor of *CEBPD* gene to **upregulate** its expression	Macrophages, Renal fibroblasts	[46, 125, 126]
	CREB	Binds to the promotor of *CEBPD* gene to **upregulate** its expression	Macrophages	[20]
	CRTC2/3	Binds to the promotor of *CEBPD* gene to **upregulate** its expression	Adipocytes, MEFs	[127, 128]
	CTCF	Binds to the promotor of *CEBPD* gene to **upregulate** its expression	Adipocytes	[124]
	CUL4B	Directly binds to the promoter of *CEBPD* gene to epigenetically **repress** its transcription and expression	Mesenchymal stem cells	[129]
	eIF4E	Binds to the promotor of *CEBPD* gene to **upregulate** its expression	MEFs	[130]
	FOXO1	Binds to the promotor of *CEBPD* gene by itself or forming a complex with CEBPD to **upregulate** its expression	Muscle cells	[48]
	GR	Binds to the promotor of *CEBPD* gene to **upregulate** its expression	Adipocytes	[131]
	HIF1a	Binds to the promotor of *CEBPD* gene to **upregulate** its expression	Glioblastoma cells	[132]
	HIF2a	Binds to the promotor of *CEBPD* gene to **upregulate** its expression	Glioblastoma cells	[132]
	JDP2	Directly binds to the promoter of *CEBPD* gene to epigenetically **repress** its transcription and expression	MEFs	[130]
	MAX	Forms the complex with MYC to binds the promotor of *CEBPD* gene for **inhibiting** its expression	Mammary, epithelial cells	[133]
	MDM2	Facilitates the assembly of the transcriptional complex consisting of CREB, Crtc2, and p300/CBP on the *CEBPD* promotor to **upregulate** its expression	Adipocytes, MEFs	[127, 128]
	MYC	Binds to the promotor of *CEBPD* gene to **inhibit** its expression	Bone marrow cells, Mammary, epithelial cells	[133–135]
	NCOA1	Forms the complex with STAT3 to binds the promotor of *CEBPD* gene for **upregulating** its expression	Mammary, epithelial cells	[135]
	NF-κB (REL)	Binds to the promotor of *CEBPD* gene to **upregulate** its expression	Macrophages, Murine embryonic fibroblasts, Hepatocytes	[123, 136, 137]
	p63	Binds to the promotor of *CEBPD* gene to **inhibit** its expression	Keratinocytes	[80]
	PAGR1	Binds to the promotor of *CEBPD* gene to **upregulate** its expression	Adipocytes	[138]
	SOX9	Binds to the promotor of *CEBPD* gene to **inhibit** its expression	MEFs	[36]
	SP1	Binds to the promotor of *CEBPD* gene to **upregulate** its expression	Macrophages, Mammary, epithelial cells	[125, 126, 135]
	STAT3	Binds to the promotor of *CEBPD* gene to **upregulate** its expression	Muscle cells Cardiomyocytes Adipocytes, Mammary, epithelial cells, Gallbladder epithelial cells	[26, 34, 35, 127, 135, 139–141]
	TET3	Binds to the promotor of *CEBPD* gene to **upregulate** its expression	Fibroblasts	[142]
**mRNA stability**	HuR	Binds to the 3′-UTR of *CEBPD* mRNA to **upregulate** its protein expression	Macrophages Mammary, epithelial cells	[38, 143, 144]
	IMP2	Binds to *CEBPD* mRNA and mediates post-transcriptional regulation to **upregulate** its protein expression	MEFs, HK-2	[143]
	PUM2	Binds to the *CEBPD* mRNA to **inhibit** its protein expression	Glioma cells	[39]
	let-7c	Binds to the 3′-UTR of *CEBPD* mRNA to **inhibit** its protein expression	Macrophages	[40, 118, 145]
	let-7-5p	Binds to the 3′-UTR of *CEBPD* mRNA to **inhibit** its protein expression	Macrophages	[41]
	miR-22-3p	Binds to the 3′-UTR of *CEBPD* mRNA to **inhibit** its protein expression	Osteoblasts	[42]
**Protein degradation**	COP1	Interacts with CEBPD for ubiquitin-mediated degradation to **inhibit** its protein expression	Mammary epithelial cells	[44]
	FBXW7α	Interacts with CEBPD for ubiquitin-mediated degradation to **inhibit** its protein expression	Macrophages	[60]
	SIAH2	Interacts with CEBPD for ubiquitin-mediated degradation to **inhibit** its protein expression	Mammary epithelial cells	[33]
	TRIB2	Serves as substrate adaptor for COP1 binding CEBPD to **inhibit** its protein expression by ubiquitin-mediated degradation	Mammary epithelial cells	[44]
	TRIM26	Interacts with CEBPD for ubiquitin-mediated degradation to **inhibit** its protein expression	Hepatocytes	[45]

### Post-translational modifications (PTMs) of CEBPD

After translation, CEBPD undergoes various PTMs that affect protein localization and stability, regulate DNA binding, and modulate its interactions with transcription factors. Current research shows that 10 sites of CEBPD protein have undergone PTMs, including phosphorylation, acetylation, deacetylation, ubiquitylation, and small ubiquitin-like modifier (SUMO)-ylation (Fig. 1, Table 2). In addition, protein kinase A (PKA) mediates CEBPD regulation by PGE2 to enhance the expression of transforming growth factor β (TGFβ) receptor 3 in osteoblasts, suggesting that PKA affects the nuclear localization and activity of CEBPD by phosphorylation Ser 191 [27, 50]. Ser 191 is located in the bZIP domain of CEBPD and functions as a nuclear localization signal that mediates CEBPD translocation from the cytoplasm to the nucleus. Moreover, PKC and mitogen-activated protein kinase (MAPK) can phosphorylate CEBPD. The predicted phosphorylation sites are Ser 208 and Ser 241 for PKC and Thr 156, Ser 160, and Ser 256 for MAPK [27].

**Table 2:** Post-translational modification of CEBPD.

Site	Manners of modification	Modifying enzymes	Effects	References
**Ser 2**	N-terminal acetylation	NatB	Unknown	[146]
**Thr 49**	Phosphorylation	GSK3β	Unknown	[60]
**Ser 57**	Phosphorylation	CK2	CK2 phosphorylation does neither influence the subcellular localization of CEBPD nor its interaction with CEBPB, but CK2 phosphorylation enhances the transcriptional activity of CEBPD in HCT116 cells	[31]
**Lys 120 (K120)**	Acetylation	p300	p300 binds to the H2 helix in N-terminal of CEBPD and acetylates probably its K120 site, enhancing the transcriptional activity of CEBPD in intestinal epithelial cells	[29, 147]
	Deacetylation	HDAC1/3/4	HDAC1/3/4 binds to CEBPD to inhibit its transcriptional activity in intestinal epithelial cells, possibly due to the deacetylation of CEBPD K120 by HDAC1/3/4	[148]
	Ubiquitylation	COP1, SIAH2, TRIM26	COP1, SIAH2, or TRIM26 binds to CEBPD and ubiquitylates its K120, reducing its protein level in breast cancer cells and hepatocytes	[33, 44, 45]
	SUMOylation	SUMO1/3	SUMO1/3 binds to CEBPD and SUMOylates its K120 inhibiting its transcriptional activity in A431 cells	[147, 149]
**Thr156**	Phosphorylation	GSK3β	GSK3β binds to and phosphorylates CEBPD at Thr156 to facilitate the recognition and binding of FBXW7α to CEBPD, enhancing its ubiquitination degradation in macrophages	[60]
**Ser 160**	Phosphorylation	GSK3β	GSK3β binds to and phosphorylates CEBPD at Ser160 to facilitate the recognition and binding of FBXW7α to CEBPD, enhancing its ubiquitination degradation in macrophages	[60]
**Ser 167**	Phosphorylation	GSK3β, p38	Phosphorylation of CEBPD Ser167 by GSK3β or p38 increases the transcriptional levels of CCL2, MMP1, and MMP3 in macrophages	[32]
**Thr171**	Phosphorylation	GSK3β, p38	Phosphorylation of CEBPD Thr171 by GSK3β or p38 increases the transcriptional levels of CCL2, MMP1, and MMP3 in macrophages	[32]
**Ser 191**	Phosphorylation	TBK1	Phosphorylation of CEBPD Ser191 enhances its DNA binding capacity and transcriptional activity in osteoblasts	[27, 150]
**Ser 256**	Phosphorylation	RIP3	Unknown	[151]

Neuronal precursor cell-expressed developmentally downregulated (NEDD)-ylation is a biochemical process in which the ubiquitin-like molecule NEDD protein 8 (NEDD8) is attached to a Lys residue within a substrate protein, intricately shaping the regulation of diverse biological processes [51]. A recent study showed that NEDD8 knockdown significantly inhibited CEBPD protein levels during adipocyte differentiation, indicating that NEDDylation maintains the protein stability of CEBPD directly or indirectly [52]. However, these data do not confirm that NEDD8 binds to CEBPD in adipocytes [52]. Given that E3 ligases of NEDDylation also serve as E3 ligases for ubiquitination and that Lys 120 of CEBPD is a key site for its ubiquitylation [44, 51], NEDDylation may also occur in CEBPD in other tissues or cell types, which requires further study. In addition, other PTMs, such as lactylation, succinylation, palmitoylation, and glycosylation, play critical roles in protein functions [53]. Further investigations are needed to determine whether CEBPD undergoes these modifications and how it acts after modification.

### Functions of CEBPD

Previous studies have shown that CEBPD regulates various physiological and pathological processes, mainly by modulating downstream target genes. CEBPD expression is low under normal physiological conditions in various cells and tissues. Nevertheless, it can be rapidly induced by external stimuli [7], suggesting that CEBPD plays an important role in the inflammatory and immune responses to these stimuli. In inflammatory cells, such as monocytes and macrophages, CEBPD can upregulate the expression of inflammatory factors, including C-C motif chemokine ligand 2 (CCL2), CCL20, COX-2, C-X-C motif chemokine ligand 1 (CXCL1), IL-6, IL-10, pentraxin 3 (PTX3), S100 calcium-binding protein a8/9 (S100a8/9), and tumor necrosis factor alpha (TNFα), in response to the stimuli [20, 22, 54–59]. CEBPD also directly increases the expressions of toll-like receptor (TLR) 4, 8, and 9 in macrophages to maintain and exacerbate the inflammatory response [60–62]. Furthermore, in hepatocytes and intestinal epithelial cells, CEBPD upregulates the expression of acute-phase proteins, including alpha-1-acid glycoprotein, C-reactive protein (CRP), and serum amyloid A (SAA), which are indicators of inflammatory progression [63–65]. In addition, elevated CEBPD upregulates component C3 expression in glial cells, retinal pigment epithelial cells, and hepatocytes, which plays a central role in the complement system [66–68]. During the immune response, CEBPD directly increases cluster of differentiation (CD) 1D expression in adipocytes, which mediates the presentation of endogenous lipid antigens to natural killer T-cells, thereby regulating the adipose tissue energy metabolism and immune homeostasis [69]. Furthermore, CEBPD mediates immunosuppression by upregulating CD274 and downregulating class II major histocompatibility complex trans-activator in macrophages [70, 71].

CEBPD plays an important role in cell differentiation and proliferation. As an adipose early differentiation factor in adipose tissue, CEBPD can not only directly upregulate late adipogenesis factor CEBP alpha (CEBPA) and peroxisome proliferator-activated receptor gamma (PPARγ), PPARγ1sv, and PPARγ2 in adipocytes to promote adipocyte differentiation [72–74]. It can also regulate the expressions of these factors indirectly through early B-cell factor transcription factor 1 (EBF1) or Krüppel-like transcription factor (KLF) 5 [75, 76]. In addition, CEBPD also targets complement factor D, and TNF superfamily member 11 expression to promote the differentiation of bone marrow stromal cells into adipocytes [77, 78]. Additionally, CEBPD is involved in skin keratinocyte differentiation by directly regulating estrogen receptor 1, musculoaponeurotic fibrosarcoma bZIP transcription factor B (MAFB), and zinc finger E-box binding homeobox 1 (ZEB1) expression [79, 80]. CEBPD promotes osteoblast differentiation by directly upregulating osteocalcin and TGFβ receptor 3 expression [50, 81]. In cell proliferation, most studies have focused on tumors, showing that CEBPD promotes cell proliferation by regulating the expressions of various target genes, including aurora kinase C, growth differentiation factor 15 (GDF15), nanog homeobox (NANOG), and the Yamanaka factors (KLF4, MYC, OCT4, and SOX2) [82–85]. Moreover, CEBPD is involved in the normal or abnormal proliferation of osteoblasts, lymphatic endothelial cells, VSMCs, endometrial stromal cells, and keratinocytes by regulating its target genes [47, 79, 86–88].

**Fig. 2:**
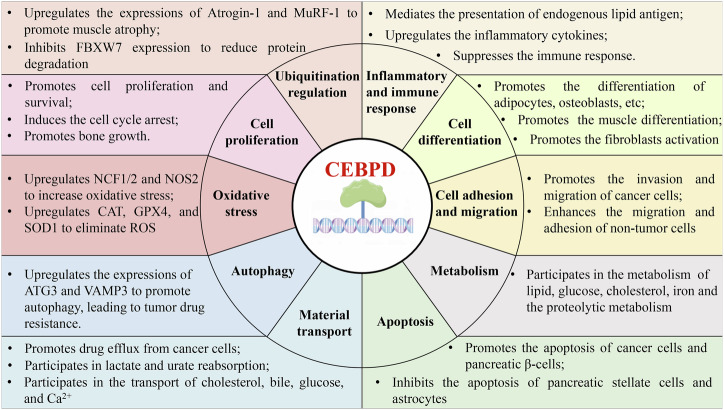
The overview of CEBPD functions.

Furthermore, CEBPD is involved in apoptosis, autophagy, cell adhesion and migration, metabolism, material transport, oxidative stress, and ubiquitination, mainly by modifying its target genes (Table 3 and Fig. 2).

**Table 3:** The target genes of CEBPD.

Types of CEBPD functions	Target genes
Regulating inflammatory and immune response (30)	AGP [63], ALOX5AP [152], C3 [66–68], CD1D [69], CD274 [70], CEBPD [46, 47], CIITA [71], COX-2 [12, 55, 153–157], CRP [64], Haptoglobin [29, 148], IKBKE [136], IL-6 [56, 59, 144, 158–161], IL6R [85], IL-8 [158, 159], IL-10 [38, 57, 162], IL23A [54], iNOS [163], KNG1 [63], LCN2 [46, 143], MCP1(CCL2) [32, 58, 164], PTX3 [20, 38, 165–169], S100a8/A9 [22], SAA [65], SFTPD [170], TLR4 [60], TLR8 [61], TLR9 [62], TNFα [59], TNFAIP6 [54, 123]
Regulating cell differentiation (26)	AMELX [171], ANGPTL4 [77], BGLAP [81, 172], CEBPA [72], CFD [77], CSF1R [173], CTH [174], CTSK [175], CTGF [19], CXCL1 [54], DLK1 [176], DSC1/3 [15], EBF1 [75], ESR1 [79], KLF5 [76], MAFB [79], MYOD1 [177], OSR2 [178], PPARG [72, 73, 179, 180], RETN [72], SREBF1 [181], TGFBR3 [50], TNFSF11 [78], TP63 [80], ZEB1 [79]
Regulating cell proliferation (21)	AURKC [82], CXCL8 [182], GDF15 [83], IGF1 [183, 184], KLF4 [85], miR-139b-3p [185], MYC [85], NANOG [84, 85], NGF [186, 187], OCT4 [84], PDGFA [188], PDGFRA [47], POU5F1 [85], PRL [88, 189], PRKDC [190], PTGIR [191], SOX2 [79, 84, 85], TGFBR2 [79], VEGFR2 [109], VEGFR3 [87], WT1 [18]
Regulating cell adhesion and migration (18)	ACTA2 [192], CCL20 [54], CCR6 [117], CTSL [193], CXCR4 [194], DSG2 [39], FN1 [132], FUT7 [117], HIF1A [87, 195], MMP1 [32], MMP2 [196], MMP3 [32, 197], MMP8 [198], MMP13 [199], RhoA [197], SDF4 [200], SNAI2 [201, 202], ST3GAL4 [117]
Regulating metabolism (14)	ADIPOQ [72, 203], AKR1C1 [204], CYP2A13 [205], CYP4A11 [21], CYP19A1 [206], GLA [69], Hepcidin [207, 208], HSD11B1 [12] LEP [72, 77, 209], LPL [72, 77], miR-429 [210], Myostatin [34, 211–213], LRH-1 [214], PSAP [69]
Regulating apoptosis (11)	CASP8 [215], CHOP [216], LC3B [217], LncRNA-Rnu3a [218], miR-135a [219, 220], miR-135a-5p [221], miR-744 [49], miR-3154 [49], miR-3162 [49], XBP1 [222], ZNF179 [223]
Regulating material transport (9)	ABCA1 [20, 84], ABCB1 [224], ABCC2 [224], ABCC3 [225], GLUT4 [193, 226], NPC2 [69], RYR1 [193], SLC5A8 [227], SLC5A12 [227]
Regulating oxidative stress (7)	CAT [228], GPX4 [229], NCF1/2 [230], NOS2 [55], NOX1 [231], SOD1 [230, 232]
Regulating ubiquitination regulation (3)	Atrogin1 [48, 212], FBXW7 [194, 210], TRIM63 [212]
Regulating autophagy (2)	ATG3 [217], VAMP3 [233]
Others (6)	ALDH1A2 [18], ET-1 [107], GNRH1 [13], miR-135a-5p [221], SCNA [234], PAI-1 [235, 236]

## Genetically modified animals of CEBPD

The knockout of CEBP family members produces different effects. CEBPA-deficient (CEBPA^−/−^), CEBP β-deficient (CEBPB^−/−^), and CEBP gamma-deficient (CEBPG^−/−^) mice showed a high mortality rate within 48 h after birth [89–91]. CEBP epsilon-knockout (CEBPE^−/−^) mice fail to produce terminally differentiated granulocytes and have a phenotype similar to that of patients with neutrophil-specific granule deficiencies [92]. However, CEBPD-deficient (CEBPD^−/−^) and CEBP zeta-knockout (CEBPZ^−/−^) mice can develop and reproduce normally [34, 90, 93], indicating that CEBPD and CEBPZ are not necessary for animals under normal conditions. Genetically modified animals with CEBPD offer the possibility of studying CEBPD functions in vivo. Although a recent study shows that CEBPD^−/−^ mice delay the onset of labor by inhibiting the expressions of COX-2 and HSD11β1 in amnion fibroblasts, which suppresses the levels of two key factors of labor onset, PGE2 and cortisol, these mice can still reproduce normally [12]. To date, the reported CEBPD-modified animals include knockout mice and zebrafish. These animals were used to study the roles of CEBPD in neurological, muscular, joint, intestinal, adipose, hematological, cardiac, and other related diseases, as well as some physiological functions (Table 4). However, all of these animals were complete knockouts. In these animals, the roles of the CEBPD knockout in one tissue, organ, or disease may be affected by its absence in other tissues and organs. Therefore, to clarify the exact functions of CEBPD in a particular tissue, organ, or disease, it is also necessary to validate them using animals with a conditional knockout of CEBPD.

**Table 4:** Genetically modified animals of CEBPD.

Modified animal type	Types of disease or physiological process	Functions	References
CEBPD^−/−^ mice	Infection	In comparison to wild-type mice, CEBPD^−/−^ mice show decreased bacterial loads in blood and brain, decreased bacterial dissemination to lung and spleen after inoculation, and lowed inflammatory mediators in the blood.	[237, 238]
CEBPD^−/−^ mice	Spinal cord injury	CEBPD^−/−^ mice show reduced glial scar formation, more residual white matter, and better motor function recovery compared with wild-type mice 28 days after the injury.	[197]
CEBPD^−/−^ mice	Alzheimer’s disease	The AppTg/CEBPD^−/−^ mice show a spatial learning improvement compared with the APPswe/PS1/E9 bigenic (AppTg) mice.	[219]
CEBPD^−/−^ mice	Alzheimer’s disease	Ablation of C/EBPD has neither in the Alzheimer’s disease model (APP/PS1double transgenic mice) nor in the prion model (scrapie-infected C57BL/6 mice) an influence on overt clinical symptoms. Moreover, the absence of C/EBPD does not affect the extent of the disease-related gliosis. However, C/EBPD-deficient APP/PS1 double transgenic mice displayed significantly increased amyloid beta plaque burdens.	[66]
CEBPD^−/−^ mice	Alzheimer’s disease	The AppTg/CEBPD^−/−^ mice show attenuated vessel formation in the brain compared with the AppTg mice.	[220]
CEBPD^−/−^ mice	Alzheimer’s disease	The AppTg/CEBPD^−/−^ mice show lowed astrocytes and microglia activation as well as increased apoptotic astrocytes in the cortex and hippocampus compared with the AppTg mice.	[32, 223]
CEBPD^−/−^ mice	Sciatic nerve crush injury	Lack of the CEBPD impairs the intrinsic capacity of peripheral neurons for regeneration	[239]
CEBPD^−/−^ mice	Experimental autoimmune encephalomyelitis	CEBPD^−/−^ mice exhibit less severe clinical disease than wild-type littermates in response to induction of experimental autoimmune encephalomyelitis.	[162]
CEBPD^−/−^ mice	Lewis lung carcinoma	CEBPD knockout in mice decreases the expression of MAFbx/Atrogin-1 and myostatin, while increasing muscle mass and grip strength.	[212]
CEBPD^−/−^ mice	Chronic kidney disease	CEBPD knockout prevents the losses of body and muscle and improves survival in CKD mice.	[34]
CEBPD^−/−^ mice	Rheumatoid arthritis	CEBPD^−/−^ mice show decreased collagen-induced arthritis score, reduced number of affected paws, and decreased pannus proliferation and angiogenesis compared with wild-type mice.	[54]
CEBPD^−/−^ mice	Radiation sickness	Irradiated CEBPD^−/−^ mice show decreased villous height, crypt depth, crypt to villi ratio and lowed expression of the proliferation marker, proliferating cell nuclear antigen, and upregulated Claudin-2 that correlated with increased intestinal permeability compared with WT mice.	[240]
CEBPD^−/−^ mice	Radiation sickness	CEBPD^−/−^ mice display increased mortality to ionizing radiation due to injury to the hematopoietic and intestinal tissues.	[241, 242]
CEBPD^−/−^ mice	Acute lung injury	CEBPD^−/−^ mice display significant attenuation of the lung permeability index, lung neutrophil accumulation, and neutrophils in bronchial alveolar lavage fluids compared with wild-type mice after intrapulmonary deposition of lipopolysaccharide.	[243]
CEBPD^−/−^ mice	Pulmonary infection	CEBPD^−/−^ mice show higher mortality induced by Klebsiella, increased bacterial loads later during infection, and decreased macrophage numbers in lungs compared with wild-type mice.	[244]
CEBPD^−/−^ mice	Lewis lung carcinoma	Deletion of CEBPD in mice results in a significant reduction of lymphangiogenesis and pulmonary metastases, with a dramatic reduction of VEGF-C and its cognate receptor VEGFR3 in lymphatic endothelial cells.	[87]
CEBPD^−/−^ mice	Acute lung injury	CEBPD^−/−^ mice show decreased S100a8/A9 expression and reduced neutrophil recruitment and cytokine release in acute lung inflammation compared with wild-type mice.	[22]
CEBPD^−/−^ mice	Inflammation	The macrophages of CEBPD^−/−^ mice show decreased IL-6 and TLR4 expression under lipopolysaccharide treatment.	[60, 123]
CEBPD^−/−^ mice	Lewis lung carcinoma	Deletion of CEBPD in mice significantly impairs myeloid derived suppressor cell expansion in response to tumor progression.	[109]
CEBPD^−/−^ mice	Disseminated intravascular coagulation	CEBPD^−/−^ mice show decreased endotoxin-induced systemic inflammation, reduced disseminated intravascular coagulation induced mortality, and improved renal function compared with wild-type mice.	[94]
CEBPD^−/−^ mice	Chronic obstructive nephropathy	Deletion of CEBPD in mice results in a more profound fibrotic response as evident from enhanced tubular injury, collagen deposition in the interstitial area, and higher expression of transforming growth factor-β.	[245]
CEBPD^−/−^ mice	Urinary tract infection	Compared with wild-type mice, CEBPD^−/−^ mice show no significant difference in bacterial clearance after urinary tract infection, no effect on the levels of inflammatory factor and chemokine production, and no effect on the extent of neutrophil infiltration in the kidney.	[246]
CEBPD^−/−^ mice	Mesangioproliferative glomerulonephritis	CEBPD^−/−^ mice show significantly less α-SMA expression in the glomerular area and less renal functional deterioration compared with wild-type mice.	[192]
CEBPD^−/−^ mice	Pancreatic ductal adenocarcinoma	Ablation of C/EBPδ does not significantly affect primary tumor growth but has a strong impact on metastases.	[247]
CEBPD^−/−^ mice	Breast cancer	CEBPD^−/−^ mice show decreased metastasis and growth of cancer cells compared with wild-type mice.	[168]
CEBPD^−/−^ mice	Breast cancer	Deletion of CEBPD increases mammary tumor multiplicity and decreases lung metastasis.	[194]
CEBPD^−/−^ mice	Skin tumor	CEBPD^−/−^ mice are not resistant to 7,12 dimethylbenz[a]anthracene -induced skin tumorigenesis.	[248]
CEBPD^−/−^ zebrafish	Cardiac injury	Knockout of CEBPD in zebrafish alters both local and distant cardiac injury responses, altering the cycling of epicardial cells as well as exchange between distant fluid compartments	[17]
CEBPD^−/−^ mice	Kidney excretion	Deletion of CEBPD in mice results in ablating the renal expression of both Na+/lactate co-transporters SLC5A8 and SLC5A12, and a marked increase in urinary excretion of lactate as well as a decrease in blood levels of lactate.	[227]
CEBPD^−/−^ mice	Neurofactor expression	CEBPD^−/−^ mice show complete loss of nerve growth factor induction in the cerebral cortex following clenbuterol treatment.	[187]
CEBPD^−/−^ mice	Mammary gland involution	In the absence of CEBPD in mammary gland, involution is delayed, and the pro-apoptotic genes encoding p53, BAK, IGFBP5 and SGP2 are not activated, while the anti-apoptotic genes coding for BFL1 and Cyclin D1 are not repressed.	[139]
CEBPD^−/−^ mice	Ovary function	Deletion of CEBPD in mice has no overt effect on ovarian physiology and reproductive function.	[249]
CEBPD^−/−^ mice	Parturition	Knockout of CEBPD in mice delays the onset of labor further supporting the concept.	[12]

**Fig. 3:**
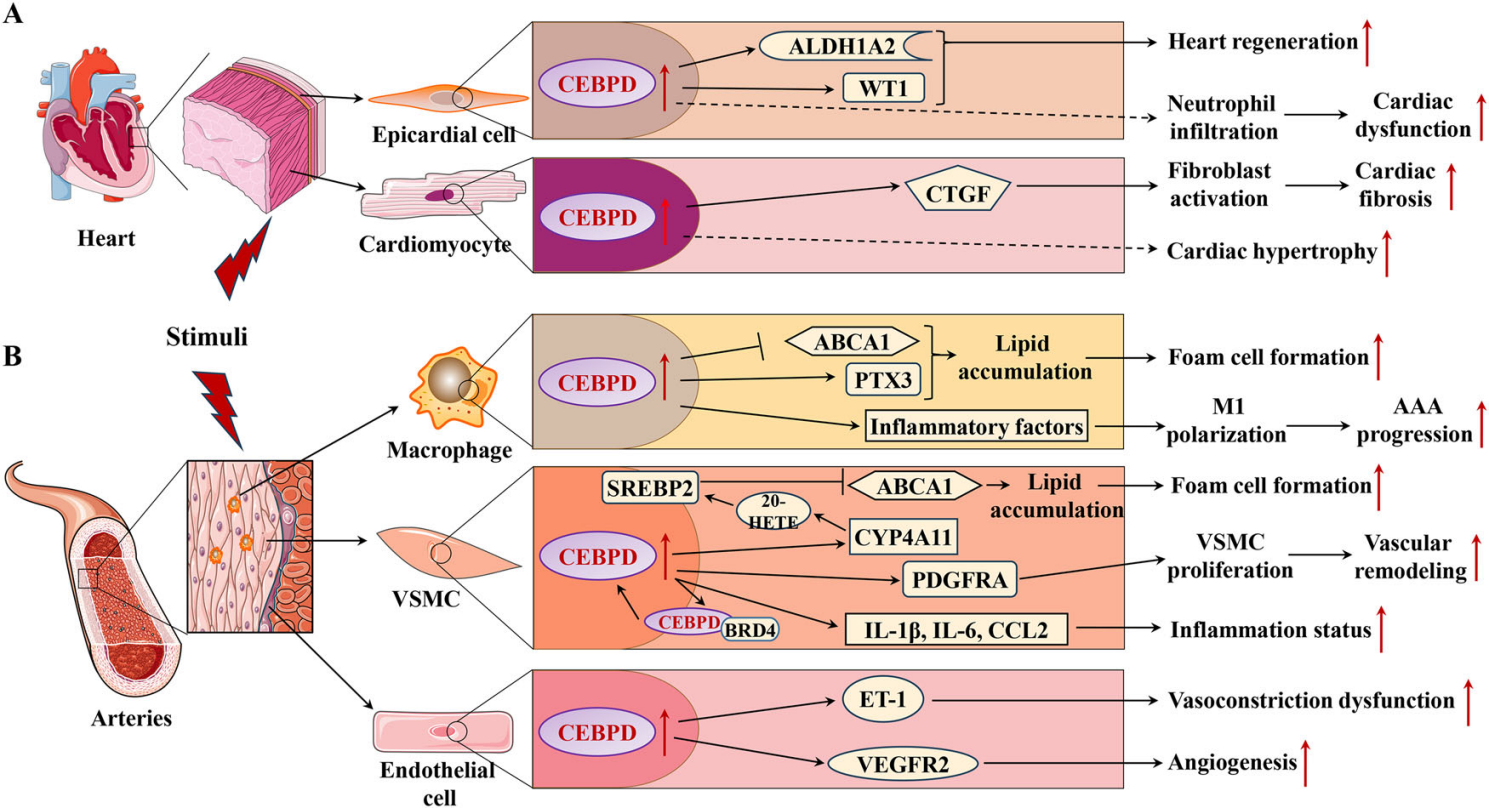
The roles of CEBPD in cardiovascular diseases. **A** The roles of CEBPD in heart diseases; **B** The roles of CEBPD in heart diseases vascular diseases.  Promotion;  Inhibition;  Indirect effect. AAA: Abdominal aortic aneurysm; ABCA1: ATP-binding cassette subfamily A member 1; ALDH1A2: Aldehyde dehydrogenase 1 family member A2; BRD4: bromodomain protein 4; CCL2: C-C motif chemokine ligand 2; CTGF: Connective tissue growth factor; CYP4A11: cytochrome P450 epoxygenase 4A11; ET-1: Endothelin 1; IL-1β: Interleukin 1 beta; IL-6: Interleukin 6; M1: M1 type of macrophage; PDGFRA: Platelet derived growth factor receptor alpha; PTX3: Pentraxin 3; SREBP2: Sterol regulatory element-binding protein 2; VEGFR2: Vascular endothelial growth factor receptor 2; VSMC: Vascular smooth muscle cell; WT1: Wilms tumor 1 transcription factor; 20-HETE: 20-hydroxy-5, 8, 11, 14-eicosatetraenoic acid.

## Roles of CEBPD in cardiovascular diseases

Under normal conditions, CEBPD knockout animals develop normally, suggesting that CEBPD has no significant effect on the development of organs, including heart and blood vessels [17, 94]. Cardiac hypertrophy caused by physiological conditions (e.g., pregnancy) does not affect the expression of CEBPD in cardiac tissue [95]. In contrast, various injury stimuli, such as transverse aortic constriction (TAC), MI, and dyslipidemia can significantly induce CEBPD expression in myocardial and vascular tissues [17–19, 21], suggesting that CEBPD mainly responds to pathological stimuli. In cardiovascular tissues, parenchymal cells (including cardiomyocytes and VSMCs) and nonparenchymal cells (such as immune cells, epicardial cells, and endothelial cells) play crucial roles in the physiological and pathological processes of heart and blood vessels. Various studies have shown that CEBPD expression in cardiomyocytes, epicardial cells, macrophages, VSMCs, and endothelial cells plays vital roles in cardiovascular diseases (Fig. 3).

### The roles of epicardial CEBPD

Recent studies have shown that CEBPD in the epicardial tissue plays a critical role in post-injury heart repair [17, 18]. CEBPD expression is upregulated in epicardial cells of the heart after ventricular apicoectomy and MI, promoting zebrafish heart regeneration by upregulating its target genes aldehyde dehydrogenase 1 family, member A2 (ALDH1A2) and Wilms tumor 1 (WT1) transcription factor [17, 18]. However, elevated epicardial CEBPD caused by MI facilitates neutrophil infiltration into the injured areas, aggravating MI-induced mouse cardiac damage [18].

### The roles of cardiomyocyte CEBPD

In addition, the CEBPD expression is increased in myocardial tissue under TAC, which is mediated by Ras-related associated with diabetes protein inhibition [19]. Furthermore, elevated CEBPD levels upregulate the expression of CTGF in cardiomyocytes to activate cardiac fibroblasts, leading to cardiac fibrosis post-TAC [19]. CEBPD protein levels can be induced by IL-6 and are mediated by STAT3 in cardiomyocytes, leading to cardiac hypertrophy via multiple mechanisms [35, 96]. These studies suggest that sustained elevated CEBPD levels in mammalian hearts exacerbate stimuli-induced cardiac injury and adverse remodeling.

### The roles of macrophage CEBPD

Accumulation of foam cells in the subendothelial space of the affected artery is a key process in atherosclerosis. Macrophages play an important role in the progression of atherosclerosis and are a crucial source of foam cells [97]. A recent study showed that CEBPD protein co-localizes with macrophages in human and mouse atherosclerotic plaques [20]. CEBPD is induced by modified low-density lipoprotein via the MAPK pathway in macrophages and promotes lipid accumulation in M1 macrophages but not in M2 macrophages [20]. Further research has shown that elevated CEBPD increases the lipid accumulation in M1 macrophages via two pathways: (1) upregulation of PTX3, which promotes the macropinocytosis of low-density lipoprotein, and (2) downregulation of ATP-binding cassette subfamily A member 1 (ABCA1) expression, which impairs intracellular cholesterol efflux in M1 macrophages [20]. In addition, macrophage CEBPD plays a crucial role in the initial inflammatory phase, which dominates the key pathogenesis of abdominal aortic aneurysm. A recent report showed that CEBPD is significantly upregulated in abdominal aortic aneurysm and that increased CEBPD promotes macrophage polarization toward M1 and maintains the M1 inflammatory state, accelerating abdominal aortic aneurysm progression [40].

### The roles of VSMC CEBPD

VSMCs are the major cell type in the tunica media. Through dynamic cell contraction and relaxation, they regulate vascular tone and blood flow [98]. In addition, VSMCs play important roles in atherosclerosis and vessel wall remodeling. VSMCs are another vital source of foam cells in atherosclerosis [99]. Recent studies have shown that elevated CEBPD binds to the cytochrome P450 epoxygenase 4A11(CYP4A11) promoter to regulate its expression in VSMCs [21]. Furthermore, CYP4A11 and its metabolite 20-hydroxyeicosatetraenoic acid promotes the activation and nucleation of sterol regulatory element-binding protein 2 to inhibit ABCA1 expression and cholesterol efflux, which promotes the transition of VSMCs to foam cells in atherosclerosis [21]. In addition, CEBPD expression was upregulated in VSMCs of the injured carotid arteries following balloon angioplasty [47, 100]. Further studies have shown that elevated CEBPD levels form a complex with bromodomain protein 4 to bind to the CEBPD promoter for positive feedback regulation in VSMCs [47]. Upregulated CEBPD enhances the expressions of IL-1β, IL-6, and CCL2 to promote the transition of VSMCs to an inflammatory state [47, 100]. Furthermore, elevated CEBPD promotes VSMC proliferation through direct upregulation of its target platelet-derived growth factor (PDGF) receptor alpha, which plays a significant role in vascular remodeling post-injury [101, 102]. Additionally, high level of insulin upregulates CEBPD expression to increase the expression levels of inflammatory factors in VSMCs, whereas TGFβ-1 inhibits the activation of VSMCs by suppressing CEBPD expression [103, 104].

### The roles of endothelial CEBPD

Vascular endothelial cells form the innermost layer of blood vessels, where they play an essential role in the development and maintenance of a functional circulatory system and provide paracrine support to the surrounding non-vascular cells [105]. Endothelial cell dysfunction initiates various vascular diseases, including atherosclerosis [97]. Elevated inflammatory factors such as IL-1β upregulate the expression of CEBPD in human aortic endothelial cells, which leads to endothelial cell dysfunction [106]. In addition, high glucose levels induce CEBPD expression through the MAPK pathway to upregulate endothelin 1 (ET-1) expression in endothelial cells, promoting vasoconstriction and blood vessel dysfunction [107]. Furthermore, decoy oligodeoxynucleotides directed against CEBPD abolished ET-1 expression in the endothelium of rabbit carotid arteries, improving the structural and functional abnormalities of blood vessels [108]. In the tumor environment, CEBPD directly upregulates the expression of vascular endothelial growth factor receptor 2 (VEGFR2) in endothelial cells to promote angiogenesis [109].

These studies indicate that upregulation of CEBPD in cardiomyocytes, macrophages, VSMCs, and vascular endothelial cells is involved in the progression of cardiovascular diseases via different mechanisms, highlighting new avenues for the development of novel therapies targeting CEBPD to treat or alleviate cardiovascular diseases (Fig. 3).

## Diagnostic significance of CEBPD in blood cells for cardiovascular diseases

Recently, transcriptome sequencing of peripheral blood cells from patients with various diseases, including cardiovascular diseases has become an important tool for studying disease mechanisms and discovering potential diagnostic biomarkers [23, 110, 111]. CEBPD levels in peripheral blood cells are lower in patients with hypertension than in healthy individuals [111, 112]. Further studies have shown that CEBPD mRNA levels are significantly decreased in the peripheral blood cells of patients who are insensitive to thiazide diuretics compared with patients who are hypertensive and sensitive to thiazide diuretics [113]. These studies suggest that CEBPD levels in peripheral blood cells negatively correlate with the progression of hypertension. Similarly, the mRNA level of CEBPD in the peripheral blood cells of patients with atherosclerosis is inversely correlated with carotid intima-media thickness, a biomarker of subclinical atherosclerosis and a predictor of future cardiovascular events [114]. This finding indicates that CEBPD levels in the peripheral blood cells are negatively correlated with atherosclerosis progression. In addition, compared with patients with type 2 diabetes and adequate glycemic control, those with type 2 diabetes and poor glycemic control have decreased CEBPD levels in the peripheral blood cells [115]. These studies suggest that peripheral blood CEBPD levels negatively correlate with the progression of chronic diseases (hypertension, atherosclerosis, and type 2 diabetes) and are potential biomarkers for these diseases.

However, CEBPD levels are significantly increased in the peripheral monocytes of patients with MI compared with those in healthy individuals [22]. Furthermore, CEBPD mRNA levels are elevated in the peripheral blood mononuclear cells of patients with acute MI compared with those in patients with stable CAD [23]. Moreover, CEBPD expression shows a stronger association with classical monocytes (CD14^++^CD16^−^) than with intermediate (CD14^++^CD16^+^) or non-classical monocytes (CD14^+^CD16^++^) [22]. In addition, CEBPD mRNA levels are higher in the peripheral blood mononuclear cells in patients with active Behçet’s disease (multisystem autoimmune relapsing vasculitis) than those in healthy individuals [116]. These reports indicate that CEBPD levels in the peripheral monocytes are positively associated with the acute state of various diseases.

These studies suggest that CEBPD in the peripheral blood is a potential biomarker of cardiovascular disease progression. However, before applying peripheral blood CEBPD as a clinical diagnostic biomarker for disease progression, more research is needed to address the following important questions: (1) What are the expression levels of CEBPD in different cell types in the peripheral blood of healthy individuals? Some reports show that CEBPD is expressed in monocytes, neutrophils, and mucosal-associated invariant T cells [22, 117]. However, the relative and absolute quantitative levels of CEBPD in different types of blood cells remain unclear. (2) Are changes in the peripheral blood CEBPD levels restricted to cardiovascular diseases? Changes in the peripheral blood CEBPD levels have also been observed in patients with other diseases. Most available research data are derived from cardiovascular diseases. Further studies are needed to investigate the changes in peripheral blood CEBPD levels in other diseases, such as liver, kidney, and lung diseases. (3) How do CEBPD levels change in different blood cells under different states of the same cardiovascular disease? For example, CEBPD levels in the peripheral blood mononuclear cells are significantly elevated during the acute phase of MI in CAD compared with that in the stable phase [23]. However, changes in CEBPD levels in the peripheral blood cells of patients with stable CAD compared with healthy individuals remain unclear, and the changes in CEBPD in different peripheral blood cell types during the stable and acute phases of CAD are unknown.

## Therapy strategies targeting CEBPD in cardiovascular diseases

Given that elevated CEBPD levels play an important role in the pathogenesis of cardiovascular diseases, suppressing CEBPD expression or activity is a promising strategy for treating these diseases. Multiple mechanisms, including transcriptional regulations, PTMs, and protein degradation, can reduce CEBPD protein levels or function. However, these processes involve various proteins essential to maintain normal cellular function and are difficult to regulate. There are two potential ways to inhibit CEBPD expression or activity: targeting CEBPD mRNA to reduce its protein levels or inhibiting its transcriptional activity using a dominant negative decoy peptide (DNDP) to bind to its bZIP domain. Several studies have shown that miR-22-3p, let-7c, and let-7-5p bind to the 3’-UTR of CEBPD mRNA in osteoblasts and macrophages to suppress its protein expression [40–42, 118]. These miRNAs target not only CEBPD but also other proteins [119–121]. Therefore, in the future, it will be necessary to predict and design miRNAs that target CEBPD with higher specificity using multiple tools, including artificial intelligence. In addition, disruption of CEBP signaling through DNDP in the adult epicardium reduces MI-induced neutrophil infiltration and improves cardiac function [18].

Furthermore, four DNDPs targeting CEBPD (CP-DN-ATF5, Bpep, Dpep, and ST101), in which dominant-negative sequences are joined with cell-penetrating domains, create drugs that can pass through tissue barriers and enter cells [122]. These peptide drugs have shown efficacy and safety in inhibiting cancer growth and survival in vivo. ST101 is currently in clinical trials for solid tumors [122]. Given that the CEBPD levels are elevated in various diseases, including cardiovascular disease, DNDPs could represent promising drugs for treating these non-tumor diseases.

## Summary and perspectives

In this review, we summarized a broad range of recent advances in research on the transcription factor CEBPD and its roles in cardiovascular disease. Recent studies have revealed much about the biology of CEBPD, particularly in various cell types. We also have discussed the versatile nature of CEBPD, which can activate or inhibit its target genes to promote many different cellular functions that affect intracellular functions or the microenvironment. These studies have partially revealed the crucial and complex role of CEBPD in the onset and progression of cardiovascular disease. Modulation of CEBPD activity through DNDPs can exert antitumor effects in the brain. Therefore, new approaches targeting CEBPD for the treatment of cardiovascular diseases have attracted attention.

Despite the encouraging progress in exploring the relationship between CEBPD and cardiovascular disease, many critical questions remain unanswered before CEBPD can be clinically utilized for cardiovascular disease treatment. It is well established that PTMs of proteins are crucial in the precise function and correct response of cells to external stimuli; however, these PTMs affect CEBPD function in cell-type-specific manner and in cardiovascular diseases remains unknown. Additionally, the knockout of highly expressed CEBPD in lymphocytes does not affect the expression of other CEBP family members [117], suggesting that, at least in lymphocytes, other CEBP family members do not functionally compensate for the absence of CEBPD. However, 35% of CEBPB^−/−^ mice and 85% of CEBPB^−/−^; CEBPD^−/−^ mice die soon after birth, indicating that CEBPD and CEBPB are interchangeable to some degree [90]. In the heart and vascular tissues, do functional substitutions exist between other CEBP family members and CEBPD? If so, how much of the functionality of CEBPD is compensated for by other members? Furthermore, although evidence shows that CEBPD levels are elevated in cardiovascular disease, the clear expression patterns of CEBPD in cardiac and vascular tissues during different disease states remain unknown. Fourth, most available studies were conducted in animal models of cardiovascular disease rather than in clinical specimens from patients with cardiovascular disease. Definitive clinical evidence of the relationship between CEBPD and cardiovascular disease is necessary for research targeting CEBPD in the treatment of cardiovascular disease. The evidence summarized in this review strengthens the hypothesis that CEBPD may be an effective target for the treatment of cardiovascular diseases and serves as a reference for further investigation in this field.

## Data Availability

All data generated or analyzed during this study are included in this published article or available from the corresponding author on reasonable request.
